# Impact of pneumonia and lung cancer on mortality of women with hypertension

**DOI:** 10.1038/s41598-016-0023-2

**Published:** 2016-12-21

**Authors:** Yuechun Shen, Zuojun Tian, Dongfeng Lu, Junyao Huang, Zuopeng Zhang, Xinchun Li, Jun Li

**Affiliations:** 1 Departments of Cardiology, The First Affiliated Hospital of Guangzhou Medical University, Guangzhou, Guangdong P. R. China; 2 Departments of Neurology, The First Affiliated Hospital of Guangzhou Medical University, Guangzhou, Guangdong P. R. China; 3 Departments of Statistics, The First Affiliated Hospital of Guangzhou Medical University, Guangzhou, Guangdong P. R. China; 4 Departments of Emergency, The First Affiliated Hospital of Guangzhou Medical University, Guangzhou, Guangdong P. R. China; 5 Departments of Radiology, The First Affiliated Hospital of Guangzhou Medical University, Guangzhou, Guangdong P. R. China; 6 Departments of General Surgery, The First Affiliated Hospital of Guangzhou Medical University, Guangzhou, Guangdong P. R. China

## Abstract

Essential hypertension is one of the most severe women’s health problems. Modern life brings more chances of pulmonary diseases to human. The purpose of the study is to investigate weather pneumonia and lung cancer are associated with the mortality of women with hypertension in different age. A cross-sectional retrospective study was conducted in women with hypertension, who were admitted into our hospital in 2004–2013. 14219 women were enrolled and 68.8 ± 12.2 year old (y). Isolated hypertension was 14.7%. The age of death was 78.1 ± 9.8 y. The mortality was 4.4% for average and 0.2%, 1.1%, 2.4%, 4.8%, 10.4% and 15.8% in group age ≦49, 50–59, 60–69, 70–79, 80–89 and ≧90 y separately. This mortality increased with age was positively significantly correlated with the increased incidences of pneumonia (*P* < 0.05, r = 0.77). Pneumonia was a significant risk associated with the mortality in age 55–89 y (OR = 6.4–22.5; 95% CI = 3.06–51.12). While, lung cancer was the significant risk in 70–79 y. These observations indicate that pneumonia and lung cancer are significant risk factors associated with the mortality of certain age women with hypertension, and bring an alert that pneumonia and lung cancer should be prevented and treated intensively in modern life in order to reduce the mortality.

## Introduction

Hypertension is one of the most severe health problems for women. The prevalence of it in women is almost the same as man, women vs. men = 26.1–29.5 % vs. 26.6–29.0%^1^. The mortality of it from 2000 through 2013 significantly increased 36.8% for aged 45–64, from 2000 to 2005 increased 23% for those >85 and 10.9% for those 75–84^2^. Women face big challenges to reduce the mortality of hypertension.

The most significant distinction between women and men with hypertension is protective effect of estrogen before menopause and reduced protective effect of estrogen after menopause^3^; therefore, women have unique manifestation of cardiovascular risks and target organ damage with hypertension, they tend to more have relevant age-related risks of mortality. Studying such risks from different age is probably powerful than studying global risks in whole age.

Pneumonia is a common disease occurring in all parts of the world^4^. It is among the most common infectious diseases and the most common lower respiratory tract infection^5^. It is a major cause of death among all age groups resulting in 7% of the world’s total death yearly^4, 6^. Lung cancer is also common worldwide, it is the third highest incidence, and second after breast cancer in mortality among cancer of women^7^. Both pneumonia and lung cancer are diseases of respiratory system, and diseases related to longevity or civilization, that appear to the increase in frequency in modern life as countries become more industrialized and people live longer.

Relationship between pneumonia or lung cancer and essential hypertension is not well studied. Pneumonia and lung cancer whether influence the mortality of patients with hypertension lacks of concrete data. So far, only few studies involved in such issue: such as, severe hypertension is commonly accompanied with pneumonia in elderly patients with acute ischemic stroke^2^. Hypertension can coexist with lung cancer, resulting in increase the risk of venous thromboembolism, which may be mediated by the presence of inflammation. The impact of pneumonia and lung cancer on mortality of women with essential hypertension needs to be identified.

Based on above information, we hypothesis that pneumonia and lung cancer may influence the mortality of women with hypertension in different age. The purpose of the study is to identify risks factors (complications and comorbidities) associated with the mortality of women with hypertension in different age, especially whether or not pneumonia and/or lung cancer belong/belongs to such factors, in order to provide evidence for establishing health education and health improving model.

The novelty and significance of the current study is that we found pneumonia and lung caner were significant risks for the mortality of women with hypertension in certain age women with hypertension, suggesting that they should be paid attention in modern life, in order to reduce the mortality.

## Patients and Methods

### Study design, settings and patients

This cross-sectional retrospective analysis was performed at our hospital. Female patients, who admitted into our hospital with hypertension between 2004 and 2013, were selected. Patients, who had secondary hypertension, repeatedly admitted (had one same admission number, only last time was retained), or lived in ineligible geographic areas prior to admission, were excluded. In order to avoid potential sources of bias and to keep subjects unified standard for living environment, eligible areas were preferred where included districts of Haizhu, Yuexiu, Liwan; The each districts had population 898,204–1,558,663, area 33.80–90.4 km^2^, and density 15,198–34,239/km^2^.

### Data collection

Data was original from databases in our hospital. Two sets of databases used were cases system and registration system of admission and discharge. Information of patients admitted into and discharged from the hospital had been officially recorded following field-specific standards for publicly available resources. The data could obtain freely in our hospital. Below information was collected for current study: name, gender, age, diagnoses, complications, comorbidities, the date of admission to and discharge from the hospital, therapeutic outcome, the cause of death, patients’ demographics, etc.

Main outcomes and measures were death, complications and comorbidities of the patients. Querying the diagnosis field according to the appropriate ICD-10 codes (The International Statistical Classification of Diseases and Related Health Problems 10th Revision).

The work did not need ethics approval. All subjects read and signed an informed consent form when they admitted into hospital. The Medical Research Ethics Committee of the Hospital reviewed and approved the present study.

### Definitions

Hypertension was diagnosed according to the diagnostic criteria of high blood pressure (BP) of WHO (The World Health Organization), that is the sustained resting either systolic BP (SBP) ≥140 mm Hg, or diastolic BP (DBP) ≥90 mm Hg, or both SBP/ DBP ≥140/90 mmg was achieved while admitting into hospital or a previous clinical record without taking antihypertensive medication.

Pneumonia was diagnosed according to comprehensive analysis of the clinical symptoms, signs, laboratory tests, tracheal aspirates cultures, chest X-ray exams and etc. It included all types of pneumonia, such as lobar pneumonia, bronchial pneumonia and acute interstitial pneumonia, caused by bacteria, viruses, and/or fungi etc. Lung cancer referred to all types of cancers arising in the lung, also included metastatic lung cancer.

Coronary heart disease (CHD) contained all types of CHD, such as acute coronary syndromes, myocardial infarction, etc. Diabetes also included abnormal glucose tolerance. Cerebral infarction (containing thrombotic infarction, embolic stroke, lacunar infarct) and cerebral hemorrhage were confirmed mostly by routine brain computed tomography (CT) or magnetic resonance imaging (MRI) examination.

### Statistical analyses

The basic data was managed by Excel software. Statistical data analyses were performed by using SPSS software (Version 17; SPSS, Inc). Continuous variables were expressed as mean ± standard deviation. Categorical variables were expressed as percentage or number. Descriptive statistics were used to describe the baseline characteristics of the patients. Mann-Whitney test was used to compare two continuous variables. Pearson Chi-square test was used to identify the association of categorical variables and mortality when <20% cells had expected count less than 5; Continuity Correction was used when ≧20% cells had expected count less than 5. Multivariate logistic backward regression models were applied to assess the associations between risk factors and mortalities. Only the results of the significant variables (*P* < 0.05) from univariate analyses were selected and entered into multivariate analysis model simultaneously. The effect of a factor was presented as the odds ratio (OR) and its 95% confidence interval (CI). *P* value of <0.05 was considered statistically significant.

## Results

### Age distribution in hospitalized women with hypertension

**Figure 1:**
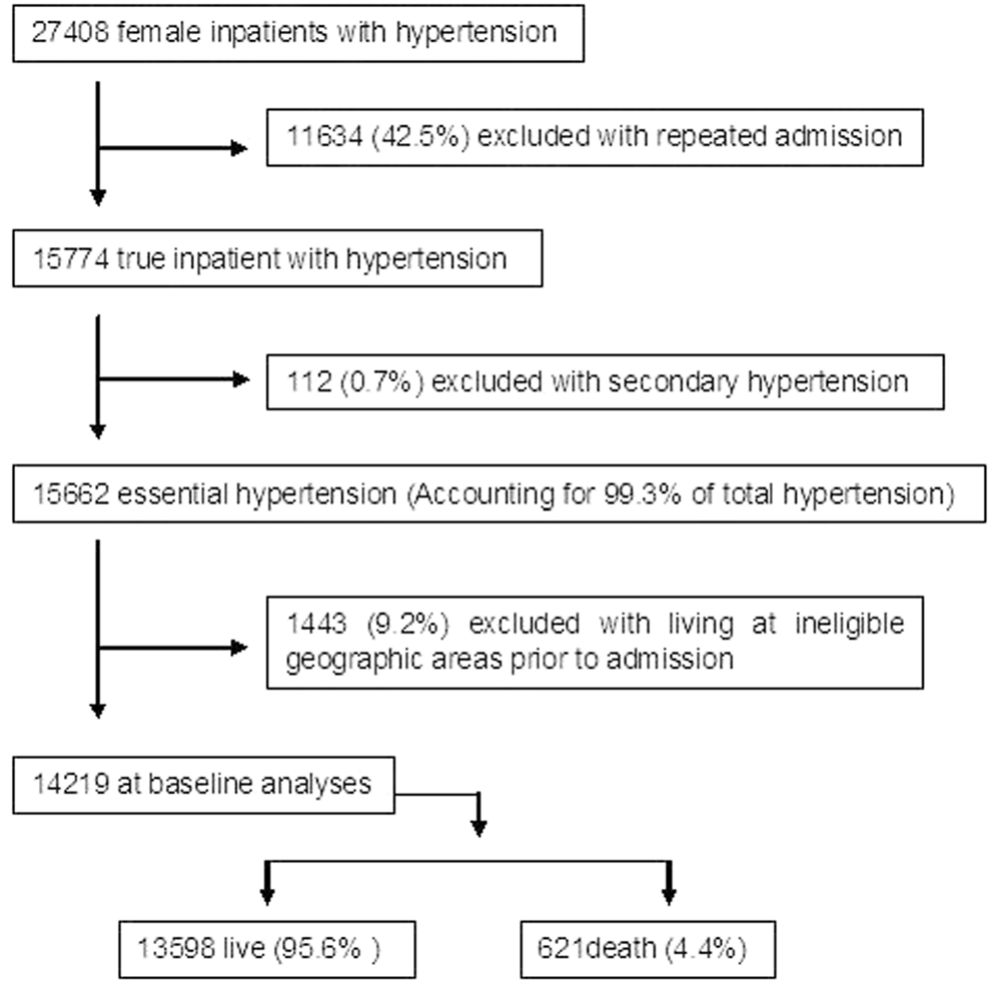
Data profile. In the end, 14219 women with hypertension were analyzed as base line in current study; among them, 4.4% was mortality.

14,219 female patients with essential hypertension during 10 year period were enrolled and used at baseline analyses (Fig. 1 and Table 1). Table 1 showed the age distribution, in which, subgroups were divided by every 5 years old (y). Mean age was 68.8 ± 12.2 y; majority of the patients was between 55–84 y (in which more than 10% in each group), accounting for 78.2%. The mean age of death was 78.1 ± 9.6 y; majority of the death was 70–89 y, accounting for 75.0%. The age group with peak percentage in both total and death was 75- group. Above observation suggested hypertension is aged disease. Trend of the numbers, % dead vs. % total in each group, is in general increased (Table 1), demonstrating the death of hypertension increased with age.

**Table 1:** Age distribution in hospitalized women with hypertension and dead ones. The data supports hypertension is an aged disease and the mortality increases with age.

Age (Years)	Total		Dead		Ratio of % (D/T)
	n	% (T)	n	% (D)	
<5	0	0	0	0	NA
5-	0	0	0	0	NA
10-	0	0	0	0	NA
15-	1	0.01	0	0	0
20-	11	0.08	0	0	0
25-	5	0.04	0	0	0
30-	35	0.25	0	0	0
35-	106	0.75	0	0	0
40-	259	1.82	0	0	0
45-	561	3.95	2	0.32	0.08
50-	1079	7.59	10	1.61	0.21
55-	1547	10.88	18	2.9	0.27
60-	1650	11.6	28	4.51	0.39
65-	1793	12.61	54	8.7	0.69
70-	2119	14.9	79	12.72	0.85
75-	2424	17.05	141	22.71	1.33
80-	1582	11.13	136	21.9	1.97
85-	774	5.44	110	17.71	3.26
90-	230	1.62	32	5.15	3.18
95-	43	0.3	11	1.77	5.90
Total	14,219	100	621	100	1.00

### Hypertensive women died most with pneumonia than other leading complications and comorbidities

**Figure 2:**
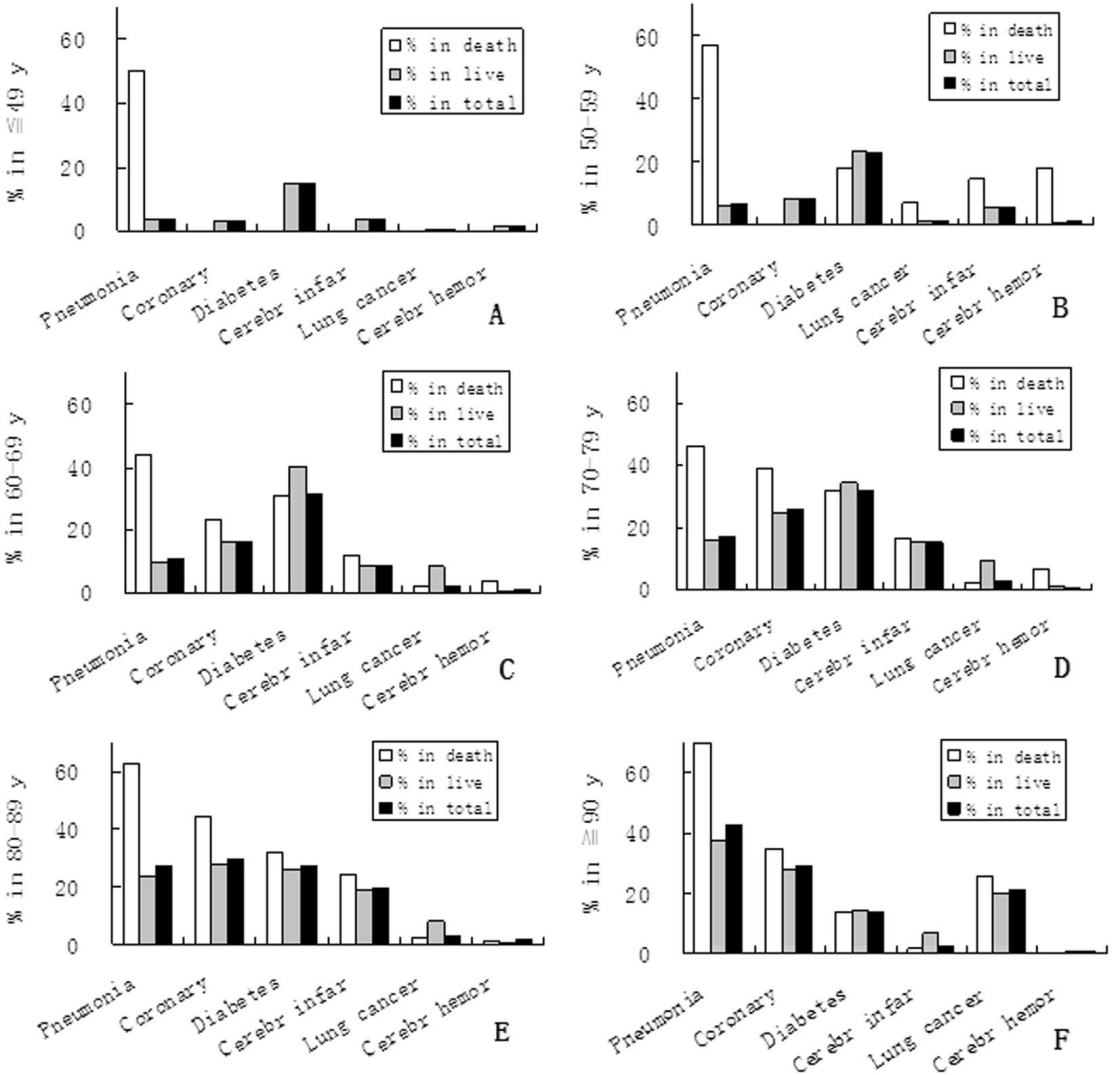
Death and incidence of pneumonia in hypertensive women in different age groups. The death rate of pneumonia (1st bar) is the highest compared with other diseases in all age groups, although arrangement positions of the incidences (2nd or 3rd bar) are different.

14.7% patient was isolated hypertension; 75.3% was accompanied by complication/s and/or comorbidity/comorbidities, including pneumonia, CHD, diabetes, lung cancer, cerebral infarction and cerebral hemorrhage (Fig. 2). Among them, the most common complications was different in different age groups: before 70 y was diabetes; in group 80–89 y was CHD; in group ≧ 90 y was pneumonia. However, the common characteristic was that the highest death rate in all groups was pneumonia. All pneumonia accompanied with other complications or/and comorbidities; no case of pneumonia was the single cause. This may be because pneumonia usually occurs in many other common illnesses especially in elderly, while hypertension is an aged disease, usually occurring in elderly also, just like our data in Table 1.

The common systems involved with diseases and their percentages in dead hypertensive women were cardio-cerebrovascular 3.1%, respiratory 2.5%, metabolic 1.7%, tumors 1.2%, digestive 0.9%, musculoskeletal 0.5% and urologic 0.4%. It’s important that the percentage of respiratory system were high, followed right after cardio-cerebrovascular; the percentage of tumors was pretty higher as well. Since pneumonia and lung caner were included in these two categories, they might play an important role in mortality of hypertension, which would be proved later in multivariate analyses of the current study.

### Univariate and multivariate analyses for risk factors associated with the mortality of women with hypertension in different age

In order to analyze factors in different age associated with the mortality, different age groups were divided and analyzed. Univariate analyses found that many factors were significantly associated with the mortality (Table 2). Multivariate analyses showed (Table 2) pneumonia was the most common risk factor in wide age with 4 groups (50–59 y, 60–69 y, 70–79 y, and 80–89 y group). In addition, lung caner was also a significant risk for the mortality in 70–79 y group, which should be related with the highest incidence of it in this compared with other groups: 0.7%, 17.7%, 21.1%, 23.6%, 19.4% and 17.4% out of 100% in groups ≦49 y, 50–59 y, 60–69 y, 70–79 y, 80–89 y and ≧ 90 y separately.

**Table 2:** Univariate and multivariate analyses for risk factors associated with mortality in different age groups of hypertensive women. Abbreviations represent: OR - odds ratio; CI - confidence interval; COPD - chronic obstructive pulmonary disease. Analyses include 7 subgroups, in which 6 are shown in the table; another one (≦49 y, n = 978, all *P* values <0.05) is not listed. Multivariate analysis is adjusted for the other factors in the table with significance (*P* < 0.05) in univariate analysis. Pneumonia and lung cancer are among significant risk factors from multivariate analyses (*P* < 0.05) in certain age groups.

Analyses	50–59 y, n = 2626			60–69 y, n = 3443			70–79 y, n = 4539			80–89 y, n = 2356			≧90 y, n = 273		
Variables (Risk factors)	Univariate	Multivariate		Univariate	Multivariate		Univariate	Multivariate		Univariate	Multivariate		Univariate	Multivariate	
	*P*	*P*	OR 95% CI	*P*	*P*	OR 95% CI	*P*	*P*	OR 95% CI	*P*	*P*	OR 95% CI	*P*	*P*	OR 95% CI
**Cardiovascular diseases**															
Coronary heart disease	>0.05			>0.05			0.000	0000	2.38	0.000	00.000	2.19	>0.05		
								1.75–3.23			1.62–2.98				
**Metabolic diseases**															
Dyslipidemia	>0.05			>0.05			>0.05			>0.05			>0.05		
Diabetes	>0.05			>0.05			>0.05			0.053	00.038	1.40	>0.05		
											1.02–1.93				
Obesity	>0.05			>0.05			>0.05			>0.05			>0.05		
**Cerebrovascular diseases**															
Cerebral infarction	>0.05			>0.05			>0.05			0.038	00.012	1.58	>0.05		
											1.11–2.25				
Cerebral hemorrhage (CH)	0.000	>0.05		0.002	>0.05		0.000	>0.05		>0.05			>0.05		
Acute CH	0.000	0.000	46.44	0.004	>0.05		0.000	0.038	6.10	>0.05			>0.05		
	12. 53–172.13							1.10–33.50							
TIA (Transient ischemic attack)	>0.05			>0.05			>0.05			>0.05			>0.05		
**Respiratory diseases**															
Tuberculosis	>0.05			>0.05			>0.05			>0.05			>0.05		
Pulmonary infection															
Pneumonia	0.000	0.000	22.58	0.000	0.000	7.17	0.000	0.000	4.72	0.000	0.000	6.40	0.000	>0.05	
		9.97–51.12			4.46–11.52			3.06–7.30			4.06–10.08				
Trachea and bronchitis	>0.05			>0.05			>0.05			>0.05			>0.05		
COPD	>0.05			0.038			0.000	>0.05		0.011			>0.05		
**Digestive system diseases**															
Upper digestive tract diseases	>0.05			>0.05			>0.05			>0.05			>0.05		
Gastrointestinal hemorrhage	>0.05			0.001	>0.05		0.000	0.008	3.30	0.000	>0.05		>0.05		
								1.37–7.94							
Gallbladder bile duct disease	>0.05			>0.05			>0.05			0.017	>0.05		>0.05		
**Tumors**	0.000			0.000	0.000	5.23	0.000	0.000	3.18	0.000	0.000	3.04	0.000		
					3.23–8.44			2.20–4.58			1.99-.4.05				
Breast cancers	>0.05			>0.05			>0.05			>0.05			>0.05		
Uterine cancer	>0.05			>0.05			>0.05			>0.05			>0.05		
Lung cancer	0.050			0.000	>0.05		0.000	0.001	2.55	0.000	>0.05		>0.05		
								1.45–4.49							
**Inflammation and infection**	0.000	>0.05		0.000	>0.05		0.000	>0.05		0.000	>0.05		0.001	0.003	3.42
														1.50–7.78	
**Acute diseases**	0.000	>0.05		0.000	0.000	4.26	0.000	0.000	3.08	0.000	0.000	2.86	>0.05		
					2.34–7.74			2.09–4.54			1.98–4.14				
**Chronic diseases**	>0.05			>0.05			0.000	>0.05		0.000	>0.05		>0.05		
**Bleeding diseases**	0.000	>0.05		0.000	0.000	7.98	0.000	>0.05		0.000	>0.05		0.000	0.000	3.51
					3.67–17.34									1.22–10.08	

Other factors associated with the mortality found in multivariate analyses were acute cerebral hemorrhage (in 50–59 y and 70–79 y), coronary heart disease (in 70–79 y and 80–89 y), and cerebral infarction (in 80–89 y) (for single disease), which are widely accepted by public.

It was worth to note that acute cerebral hemorrhage was a risk of death in age groups 50–59 and 70–79, but not in 60–69 and 80–89. This result was consistent with the dead incidence of it in each group: counting for 17.9%, 3.7%, 5.5% and 2.4% in 50–59, 60–69, 70–79 and 80–89 y separately. The phenomena may be related with blood pressure higher in earlier stage of hypertension (50–59 y, around menopause) and using of anticoagulation medication in 70–79 y. The result was similar to a report that there was no significant trend for cerebral hemorrhage due to hypertension with increasing age^8^, and explained by another report that the rate of use of antithrombotic agents increases with age, antithrombotic drugs may be one of the causes of poorer outcome in aged patients with hypertensive intracerebral hemorrhage^9^.

### Correlation of morbidity with mortality in hypertensive women

**Figure 3:**
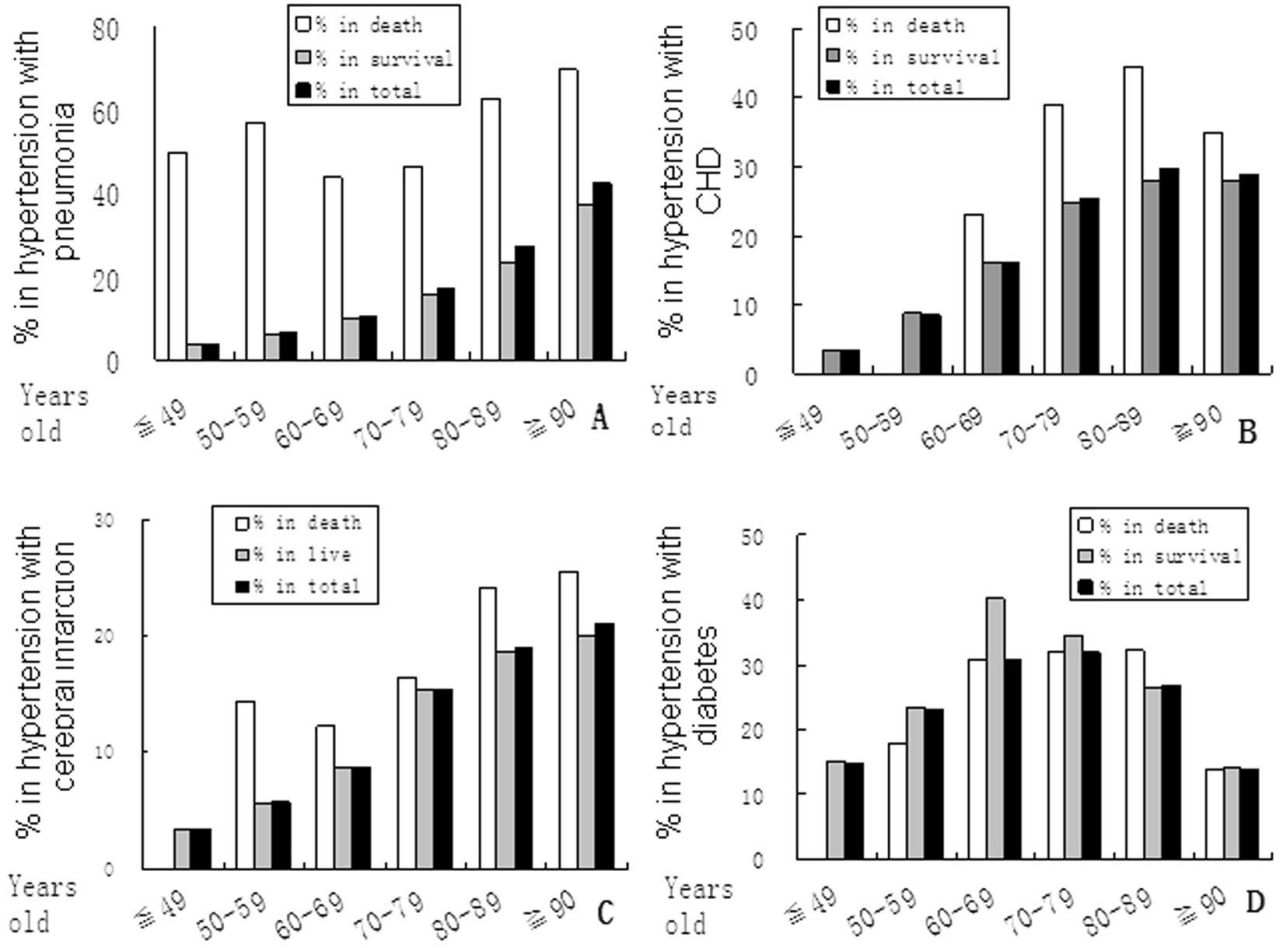
Correlation of morbidity and mortality of risks in hypertensive women. The incidences of pneumonia **(A)** increase with age (black bars); the death rates increase with age as well in general (white bars), resulting in the correlation coefficient between them significant (*P* < 0.05, r = 0.77). The correlation coefficient between incidences and mortalities in different ages from all other risks (**B**, coronary heart disease; **C**, cerebral infarction; **D**, diabetes) is not significant.

It’s reasonable but still little surprise that the incidence of pneumonia in hypertensive women increased with age (Fig. 3), and the mortality increased with age in general as well, resulting in the correlation coefficient (CC) significant between them: *P* < 0.05 = 0.002, r = 0.77. While, both the incidence and mortality of CHD increased with age but not in ≧ 90 y group; the CC was even not significant (*P* > 0.05, r = 0.98) (Fig. 3). CC was also not significant between mortality and incidence of cerebral infarction (*P* > 0.05, r = 0.90), diabetes (*P* > 0.05, r = 0.83), acute cerebral hemorrhage, and lung caner separately. Therefore, the mortality of women with hypertension increased with age was mainly because it significantly positively correlated with pneumonia.

**Figure 4:**
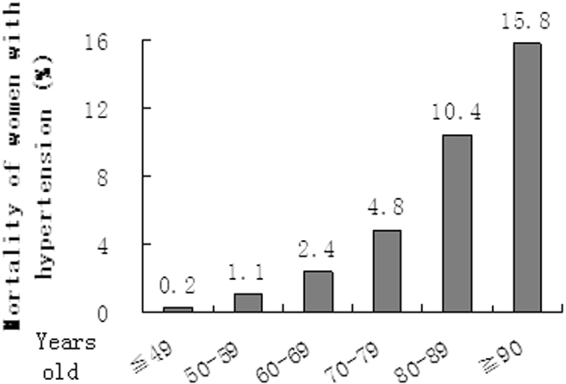
Hospitalized mortality of women with hypertension in different age groups. The mortality increases with age.

**Figure 5:**
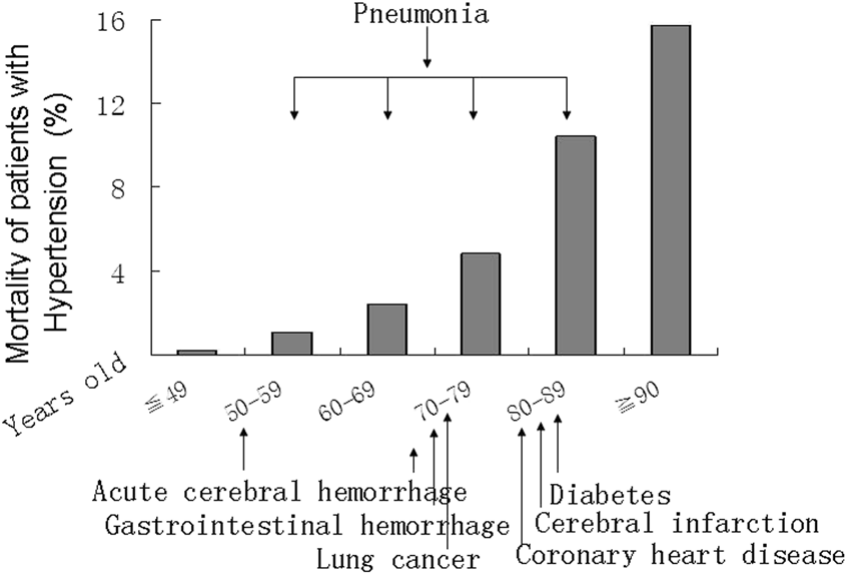
Risk factors associated with the mortality of women with hypertension in different age groups. Diseases, which are pointed in the figure, are risk factors associated with the mortality corresponding to different age groups.

The hospitalized mortality of women with hypertension increased with age (Fig. 4). The death rates were 0.2%, 1.1%, 2.4%, 4.8%, 10.4% and 15.8% in group age ≦ 49, 50–59, 60–69, 70–79, 80–89 and ≧90 y separately. The risk of mortality in group age 50–59, 60–69, 70–79, 80–89 and ≧90 y increased 4.4, 8.3, 13.4, 24.5 and 33.5 times compared with ≦49 y group orderly. The distributions of significant risk factors associated with the mortality in different age groups were shown in Fig. 5.

## Discussion

Factors associated with the mortality of hypertension are important; hence they are interested by researchers and studied much. There are some factors reported, such as cardiovascular diseases, smoking, heavy alcohol use, chronic obstructive pulmonary disease (COPD)^10^, arterial ischemic stroke^11^, diabetes^12^, serum uric acid^13^, geographic concentrations of medical doctors^14^, physical activity^14^, sex and ethnicity^15^, coronary artery calcium^16, 17^, an so on. However, to our knowledge, pneumonia and lung cancer are not reported. Nowadays, people enjoy modern life, at the same time, they lost the fresh air. Respiratory diseases have increased and affected people’s health. The impact of them on hypertension needs to be evaluated.

In current study, we performed a cross-sectional study on hospitalized women with hypertension, and have a novel founding that pneumonia was the most common risk factor associated with the mortality, not only it was significant (*P* < 0.05) in multivariate analysis and lasted over long period of age, from 50 to 89 years old, but also it was positively correlated with the mortality. This conclusion was not found directly in literature, but supported indirectly by below evidences: pneumonia ① is a common illness affecting approximately 450 million people a year and occurring in all parts of the world^18^; ② is a major cause of death among all age groups resulting in 7% of the world’s total death yearly, 10–25% in hospital death being particularly high in older adults and in patients with comorbidities^19, 20^; ③ is at a high prevalence related to cardiovascular diseases in hospital admissions and a trend toward an increased risk of an poor outcomes^21, 22^. Therefore, pneumonia is a very common disease causing significant morbidity and mortality especially in elderly and patients with cardiac complications. Coincidentally, hypertension shares these characteristics as well. Underlying condition of hypertension and pneumonia, patients should have worsening outcomes.

In addition, we found lung cancer was significant risk of mortality for 70–79 y hypertensive women, which was probably related with the characteristics of the lung cancer. Research has found^23^, lung cancer is a disease of the elderly; the mean age at the time of diagnosis is 71 years; no cases were diagnosed in patients younger than 20 years; more than 65% of patients with lung cancer are older than 65^23^. Consistent with this, we found no cases were diagnosed in patients younger than 20 years either; more than 65% (75.4%) of patients with lung cancer are equal and older than 65 as well.

One of the important reasons for above results from current study, about impotent role of pneumonia and lung cancer on mortality of women with hypertension, may be related with high population densities and heavy air pollution. The individuals enrolled in current study were all from three downtown district areas of Guangzhou. The districts had large population and high population density; the city is the third largest city in China, the largest city in South Central China and serves as an important national transportation hub and trading port. So, more mortality and morbidity of pneumonia and lung cancer should be related with adverse environmental conditions.

It was worth to note that medication for lung cancer might make essential blood pressure higher or make the outcomes worse, because studies have showed Bevacizumab, a medicine of lung cancer, is associated with a significantly increased risk of hypertension development in non-small-cell lung cancer patients^24^. Three most important adverse events of regorafenib, an another medicine of lung cancer with a multi-target inhibitor for vascular endothelial growth factor receptor, were hypertension, diarrhea, hand/foot skin reactions with potential blisters^25^. All of these effects were manageable with appropriate dose modifications^25^. Hence, clinicians should be ware of causal relationship of medication of lung cancer to hypertension, and resistant hypertension may be related with the medication. In time prevention and treatment of the side effects of medicine are significant.

Inner-link may exist among pneumonia, lung cancer and women hypertension. Major pathophysiologic mechanisms of hypertension include (1) increasing cardiovascular risk factors, such as ① diabetes, which can increase risk for pneumonia; ② stroke: about one third of patients after acute stroke has been estimated to occur pneumonia^26^; (2) vascular stiffness and endothelial dysfunction with oxidative stress, resulting arterial endothelial damage and more reactive oxygen species (ROS), which is one of the key signaling molecules that play an important role in the progression of inflammatory disorders^27^; (3) heart failure or kidney failure etc., leading to low immunity, in which respiratory tract infection will occur first rather than other system disorders. All above shows close relationship from hypertension to pneumonia. In addition, people with lung cancer have an increased risk of developing pneumonia. While, past lung diseases for example recurrent pulmonary infection may be at a greater risk of developing lung cancer^28^. These show the relationship between pneumonia and lung cancer. All in all, human body is a whole unit, one disorder may cause another disorder; especially, disorders of cardiovascular and respiratory system are always appear constantly; if one of them can not be controlled, then deadly conditions will occur.

We found ≧65 years age women with hypertension were 63.05%, which was consistent with a review that approximately 60% of women was ≧65 years^29^, but not in agreement with a report that 60% of women was <54 years in Mexico, where woman have a unique nutritional culture^30^. Our results support hypertension is an age related disease that is widely accepted.

It was reported that only 20% of women present an isolated hypertension in 2001^31^. In current study, we found it was less, as 14.7%; which may be the times of the studies different. At present, air pollution causes more respiratory diseases; cancer risks (harmful chemicals etc) increase cancer incidence; diet condition results more metabolic disorders; and medical condition makes diagnoses more completed. All of these factors could be related with less isolated hypertension. it was reported that hypertension usually occurs in conjunction with metabolically coupled risk factors, such as obesity, diabetes, high cholesterol, coronary events and stroke^31, 32^; our results show not only so, but also hypertension is usually accompanied with other comorbidities, such as pneumonia and cancer.

Many organizations including current Chinese guidelines for the management of hypertension^33^ point out that the goal for blood pressure treatment was <140/90 mm Hg, but not emphasize the comorbidities and complications of hypertension. Current results suggest that comorbidities and complications of hypertension, especially pneumonia and lung cancer should be prevented and treated intensively in order to reduce the mortality.

Limitations of the study are: Firstly, more detailed information should be provided, such as whether or not smoking, the condition of medication used for hypertension and the levels of BP (Though some people suggest that 1 or 2 times of BP can not represent the patients’ true BP). Since the study design is retrospective analysis, and the number of patients is large, we did not review case file for these information one by one. Secondly, although current study analyzed a pretty large number of hypertensive women (14219 subjects) for pretty long time (10 years), it carried out in a single center; which could not provide the advantages of multi-center studies. The results might in part represent a certain situation of hypertension at downtown areas in China; they could not refer to all situations. Finally, the findings in men were not included due to data large amount. How different effects of pneumonia and lung cancer on mortality of different age men and women with hypertension needs to be further analyzed.

In conclusion, in this study, we explored risk factors associated with the mortality of Chinese women with hypertension in a single center. We proved hypertension was an age related disease and found the death rate was increased with age, furthermore, the death rate was positively significantly correlated with the increased incidence of pneumonia. Lung cancer was also significant risk factor for mortality of women with hypertension in certain age. These observations demonstrate that except for traditional risks for mortality of women with hypertension, pneumonia and lung cancer should be paid attention in order to effectively reduce the mortality.
